# SAGES colorectal and metabolic bariatric surgery committee joint task force call to action for synchronized severe obesity and colorectal cancer management

**DOI:** 10.1007/s00464-026-12878-3

**Published:** 2026-05-18

**Authors:** Andreas M. Kaiser, Heather Carmichael, Lawrence Lee, Nathan Aminpour, Nawar A. Alkhamesi, Ana Sofia Ore, Siva Dantu, Evelyn Janet Bonilla, Teresa deBeche-Adams, Mukta K. Krane, Farah A. Husain, Omar M. Ghanem, Nour El Ghazal, Patricia Sylla, Elisabeth C. McLemore

**Affiliations:** 1 Department of Surgery, City of Hope National Medical Center, Duarte, CA USA; 2 Department of Surgery, Penn State University Hershey Medical Center, Hershey, PA USA; 3 Colon and Rectal Surgery, McGill University, Montreal, QC Canada; 4 Department of Surgery, Kaiser Permanente, Los Angeles, CA USA; 5 Kadlec Regional Medical Center, Washington State University, Richland, WA USA; 6 Department of Surgery, Mayo Clinic, Rochester, MN USA; 7 Upstate University Hospital, Syracuse, NY USA; 8 Department of Anesthesiology, City of Hope National Medical Center, Duarte, CA USA; 9 Colon and Rectal Surgery, Advent Health, Orlando, FL USA; 10 Department of Surgery, University of Washington, Seattle, WA USA; 11 Department of Surgery, Oregon Health & Science University, Portland, OR USA; 12 Department of Surgery, Mount Sinai Medical Center, New York, NY USA

**Keywords:** Obesity, Severe obesity, Colorectal cancer, Colon cancer, Rectal cancer, Perioperative risk reduction, Complications, Guidelines, Surgical strategies, Prehabilitation, Multidisciplinary cancer care

## Abstract

**Background:**

Severe obesity (body mass index ≥ 40 kg/m^2^ or ≥ 35 kg/m^2^ with obesity-related comorbidities) is increasingly prevalent and independently associated with elevated perioperative morbidity and inferior oncologic outcomes in patients with colorectal cancer (CRC). Despite these risks, intentional preoperative weight optimization is not routinely incorporated into CRC management, owing to concerns regarding treatment delay, absence of guideline endorsement, and limited supporting evidence.

**Methods:**

A literature review was conducted using PubMed and Embase to evaluate the impact of severe obesity on morbidity, mortality, and oncologic outcomes in CRC. Peer-reviewed English-language studies involving adult human subjects were included, while conference abstracts, non-English publications, and studies unrelated to obesity and CRC were excluded. In the absence of published reports describing synchronized weight loss and CRC management in patients with severe obesity, three novel retrospective case examples were included to demonstrate feasibility during neoadjuvant treatment, with institutional review board approval obtained for all cases.

**Results:**

Severe obesity complicates CRC staging due to limitations in cross-sectional imaging and anatomic delineation. Furthermore, severe and particularly visceral obesity is associated with increased rates of anastomotic leak, surgical site infection, and conversion to open surgery. Current CRC guidelines do not incorporate structured weight-loss strategies into standard treatment algorithms. Metabolic bariatric procedures, such as sleeve gastrectomy, achieve rapid and clinically meaningful weight reduction, often resulting in improved operative exposure and technical conditions for subsequent resection. Pharmacologic therapies, while more broadly accessible and less invasive, typically yield more modest reductions in visceral adiposity. Task force members report early experience across three distinct cases of locally advanced CRC in patients with severe obesity, demonstrating successful preoperative visceral fat reduction through multidisciplinary coordination incorporating metabolic bariatric surgery or pharmacologic therapy during neoadjuvant windows, followed by definitive oncologic resection.

**Conclusions:**

Severe obesity adversely influences CRC staging, operative complexity, and perioperative outcomes. Intentional metabolic optimization—through bariatric surgery or pharmacologic therapy—may represent a viable adjunct within multidisciplinary, patient-centered CRC care pathways. However, the absence of prospective short- and long-term outcome data underscores the need for systematic investigation to define optimal timing, safety parameters, and oncologic efficacy of weight-loss interventions in this high-risk population.

Obesity (body mass index [BMI] ≥ 30 kg/m^2^) and severe obesity (BMI ≥ 40 kg/m^2^) are rapidly increasing worldwide and are associated with higher cancer incidence, including colorectal cancer (CRC). Obesity is also linked to chronic comorbidities that increase operative risk and limit the efficacy of nonsurgical treatment options. Severe obesity introduces additional technical challenges in patients with CRC, including impaired imaging quality, the need for specialized equipment, and increased anesthesia-related risks such as hypoventilation, carbon dioxide retention, and reduced tolerance of pneumoperitoneum. In some patients, these factors may preclude surgical intervention altogether [1].

Obesity is defined as excess fat accumulation impairing overall health [1]. BMI-based classification includes normal weight (18.5–25 kg/m^2^), class I obesity (30–34.9 kg/m^2^), class II obesity (35–39.9 kg/m^2^), and class III or severe obesity (≥ 40 kg/m^2^) [2]. However, BMI inconsistently correlates with surgical outcomes due to individual variation in fat distribution by body type, sex, and ethnicity [3]. Alternative metrics include computed tomography (CT)-based visceral fat assessment, defining visceral obesity as a visceral fat area (VFA) > 100 cm^2^ or a visceral-to-subcutaneous fat ratio (V/S) > 0.4 [4]. Waist circumference thresholds (> 102 cm in men, > 88 cm in women) and the visceral adiposity index (VAI)—incorporating BMI, waist circumference, triglycerides, and high-density lipoprotein cholesterol—are also used [5]. Sarcopenic obesity, characterized by low skeletal muscle area and high VFA on CT imaging, has also been associated with inferior perioperative and oncologic outcomes.

Since 1990, adult obesity has more than doubled worldwide, while adolescent obesity has quadrupled [6]. Obesity is now the most prevalent chronic disease in developed countries [7]. In the United States, obesity affects 40.3% of adults and severe obesity affects 9.4% of adults [8]. Prevalence peaks between ages 40 and 59 and is lower among individuals with higher levels of education. By 2030, nearly half of U.S. adults are projected to have obesity, and one in four are projected to have severe obesity [9]. Obesity contributes to metabolic syndrome, a chronic proinflammatory and prothrombotic state, associated with hypertension, obstructive sleep apnea, type 2 diabetes, hyperlipidemia, heart failure, osteoarthritis, gastroesophageal reflux disease, and liver disease [8, 10]. A pooled analysis demonstrated a 7–14-year reduction in life expectancy among individuals with BMI 40–59.9 kg/m^2^ compared with those with BMI < 30 kg/m^2^, driven primarily by cardiovascular disease, cancer, and diabetes [11].

Obesity increases cancer risk through adipose-derived cytokines and tumor-promoting inflammation [12, 13]. Excess adiposity is linked to malignancies in 13 anatomic sites, including the colon, rectum, breast, endometrium, esophagus, and pancreas, with up to 38% attributable to obesity [12, 14]. Rising cancer incidence in younger adults has also been associated with obesity and type II diabetes, with elevated BMI in adolescence or early adulthood conferring increased cancer risk later in life [15–19]. Women with obesity experience higher cancer incidence and mortality, including CRC. Weight loss favorably modifies inflammatory and metabolic biomarkers such as C-reactive protein, tumor necrosis factor, interleukin-6, and insulin-like growth factor. Time-restricted eating and bariatric surgery have both been associated with reduced tumor initiation and lower CRC incidence [15, 20–24].

Historically, obesity management has been deferred until CRC treatment is completed to avoid any delay in initiation of oncologic therapy. However, severe obesity itself may significantly impair cancer treatment delivery and outcomes. Treatment intervals—such as neoadjuvant therapy for rectal cancer or systemic therapy for advanced colon cancer—may provide opportunities to manage obesity concurrently with CRC management. This manuscript, developed by the Society of American Gastrointestinal and Endoscopic Surgeons (SAGES) Colorectal and Metabolic and Bariatric Surgery Joint Taskforce, examines the impact of severe obesity on colorectal cancer and delineates opportunities for coordinated, multidisciplinary management. We further identify critical gaps in the integration of weight-loss strategies into patient-centered oncologic pathways, propose practical windows of therapeutic opportunity, and present early experience with synchronized metabolic optimization and CRC care to help inform and pave the way for future prospective investigations.

## Methods

A comprehensive literature review was conducted to evaluate current evidence on severe obesity, its associated complications, and its impact on morbidity, mortality, and oncologic outcomes in patients with both severe obesity and CRC. The search strategy included electronic databases PubMed and Embase from inception to January 31, 2026. Keywords and Medical Subject Headings terms included “severe obesity,” “obesity,” “colorectal cancer,” “delayed surgery for colorectal cancer,” “prehabilitation,” and “surgical outcomes.”

Inclusion criteria comprised peer-reviewed original research articles, reviews, and clinical guidelines published in English. Selected publications focused on adult human subjects and addressed obesity classification, treatment strategies, or outcomes in colorectal cancer. Exclusion criteria included conference abstracts, non-English publications, and studies unrelated to the primary topic. Titles and abstracts were screened independently by all authors. Full texts of potentially relevant articles were assessed for eligibility. Discrepancies were resolved through discussion during virtual and in-person joint task force meetings. Data extraction included study design, population characteristics, obesity measures, interventions, and key findings. Due to heterogeneity in study designs and outcomes, a qualitative summary was created.

As no published case reports or case series currently describe synchronized weight loss and colorectal cancer management in patients with severe obesity, task force members with early experience using this novel approach included three unique retrospective case examples to demonstrate the safety and feasibility of weight loss during neoadjuvant treatment. Institutional review board approval for retrospective outcomes in colorectal cancer was obtained for all cases. Artificial intelligence-based tools were used only after completion of the initial manuscript draft and were limited to editorial functions (grammar, syntax, and formatting). All scientific content, analyses, and interpretations were generated solely by the authors.

## Results

### Impact of obesity and severe obesity on colorectal cancer management

There is overwhelming evidence documenting the negative impact of obesity and severe obesity on CRC diagnosis and management. Obesity and severe obesity can lead to inaccurate clinical staging which can lead to either over or undertreatment of the underlying malignancy [25–29]. In addition, adjuvant and neoadjuvant treatment planning such as radiation and chemotherapy is also adversely impacted by obesity and severe obesity, further contributing to the potential for under or overtreatment [30–35]. Perioperative ostomy planning, risk reduction strategies, intraoperative anesthesia, and surgical techniques are also challenged in patients with obesity and severe obesity and CRC [36–83]. These challenges are outlined in greater detail below.

#### Cancer staging challenges

Disease assessment and staging of CRC rely on clinical, endoscopic, and radiographic evaluation which may include computed tomography (CT), magnetic resonance imaging (MRI), and positron emission tomography (PET). However, imaging accuracy and feasibility are compromised in patients with severe obesity. CT and MRI scanners have physical limitations in weight capacity and aperture size. Modern CT tables accommodate up to 200–300 kg, while MRI systems typically allow 160–250 kg (up to 315 kg in open MRI, but with significantly reduced imaging quality). Aperture diameters limit patient girth to 85 cm (CT) and 70 cm (MRI) [16–18].

Even when imaging is technically feasible, interpretation is challenged by field-of-view limitations in obesity (~ 50 cm). These limitations cause truncation or wrap-around artifacts in the captured images. Increased adiposity attenuates photon beams in CT, increasing noise and motion artifacts, often necessitating higher radiation doses. Open MRI suffers from inadequate fat saturation and suboptimal image quality with visceral obesity. Patient comfort and claustrophobia, especially with the MRI’s longer bore and smaller aperture, further impact image quality [18, 19, 84]. Obesity—particularly severe obesity—compromises the accuracy of staging modalities, diminishing the reliability of clinical staging and increasing the risk of both overtreatment and undertreatment of the underlying malignancy.

#### Neoadjuvant treatment difficulties

Radiotherapy is a common neoadjuvant treatment used in patients with rectal cancer. Obesity complicates radiotherapy delivery, as a large pannus can shift tattoo markings and reduce treatment reproducibility. Immobilization may be less effective, and imaging quality for dose planning and alignment is often suboptimal [85]. Despite these recognized challenges, there are currently no published studies specifically examining the impact of severe obesity on outcomes of neoadjuvant radiation therapy in rectal cancer.

Chemotherapy is central to neoadjuvant and adjuvant colorectal cancer management and may serve as a nonsurgical option for patients with limited operability [86]. Dosing is typically based on body weight or body surface area. However, reduced adipose perfusion in obesity may increase effective drug exposure in organs and tumors. To limit toxicity, clinicians often cap doses near the ideal body weight, resulting in dose reductions in up to 32% of patients with obesity [87]. Such reductions are associated with worse progression-free survival [88, 89]. Current ASCO guidelines recommend full, weight-based chemotherapy dosing regardless of BMI [90]. Consequently, obesity may also contribute to either overtreatment or undertreatment with systemic chemotherapy as well.

#### Perioperative risk assessment and outcome hurdles

Accurate perioperative risk stratification is essential for all surgical patients and is particularly important in those with severe obesity. Obesity-related comorbidities, including hypertension, diabetes, atherosclerosis, and obstructive sleep apnea, increase perioperative cardiovascular risk [91]. Severe obesity also limits diagnostic accuracy, as echocardiography, MRI, and stress testing may be compromised by poor image quality. Prehabilitation focuses on optimizing modifiable risk factors, including glycemic control. Targeting a preoperative hemoglobin A1c < 7.0% and maintaining perioperative blood glucose ≤ 110 mg/dL are associated with improved outcomes but are often more difficult to achieve in patients with obesity and severe obesity [92]. Despite its potential benefit, weight loss prehabilitation is not routinely offered to colorectal cancer patients, largely due to concerns about delaying oncologic treatment and the lack of surgical society guidelines supporting the integration of weight loss strategies with neoadjuvant CRC therapy.

#### Pre-operative ostomy marking and planning

Stoma creation is frequently an essential component of colorectal surgery and is associated with complication rates of 21–70% [93]. These risks are amplified in patients with obesity, who are more prone to parastomal hernia, stoma retraction, mucocutaneous separation, incarceration, and functional impairment [25]. Optimizing outcomes requires meticulous preoperative planning and attention to key technical factors (Table 1).

**Table 1:** Optimized stoma creation in patients with obesity

Task/benchmark	Problem	Recommended approach
Stoma marking	Patient unable to see stoma that rolls underneath the pannus	Location in upper abdomen where the abdominal wall is relatively thinner and the patient can see the stoma
Creation of trephine	Thickness of subcutaneous fat challenging to expose fascia and muscle level	Consideration of inside-out construction of stoma during MIS
Sufficient bowel reach	Thick mesentery or mesocolon with limited reach	Adequate mobilization and thinning out of non-essential fat components, possible high ligation, possible flexure mobilization, pseudo-loop stoma
Width of bowel	Large bowel diameter (e.g., as result bowel obstruction or excessive appendages	Trimming of appendages, bowel decompression prior to pulling through abdominal wall
Stoma retraction	Retracted ileostomy more difficult to manage than retracted colostomy	Rather diverting transverse colostomy than poor ileostomy
Stoma for weight loss		Slightly more proximal creation of ileostomy may allow for some weight loss (but increases risk of dehydration)

In patients with colorectal cancer and obesity, anticipation of elevated perioperative risk often favors a staged surgical strategy***.*** The staged approach may include proximal diversion with or without primary anastomosis, to mitigate morbidity and mortality associated with anastomotic leak. When adjuvant chemotherapy is indicated for high-risk colon cancer based on stage or adverse tumor biology, postoperative complications—particularly anastomotic leak or persistent infection—may substantially delay the initiation of postoperative systemic therapy.

#### Perioperative DVT and PE prophylaxis

Colorectal cancer confers a 9–12-fold increased risk of deep vein thrombosis (DVT) through tumor-mediated activation of the coagulation cascade and overexpression of procoagulant factors such as tissue factor [26]. Major abdominal or pelvic surgery, immobility, malignancy, chemotherapy, and obesity are independent risk factors for venous thromboembolism (VTE), with additive effects when combined. The reported incidence of DVT within 90 days after colorectal cancer resection is 2–6% [27]. Chemotherapy and immunotherapy further increase VTE risk [28] Obesity confers additional risk comparable to thrombophilia, exceeding 14% in severe obesity; patients with BMI ≥ 35 have a 6–sevenfold higher risk than normal-weight individuals [29, 30].

VTE prophylaxis is essential in hospitalized surgical patients to reduce the incidence of DVT and pulmonary embolism (PE) [31]. Both pharmacologic and mechanical strategies are recommended. However, pharmacologic prophylaxis carries an increased risk of major bleeding (relative risk 2.87) [32]. Low-molecular-weight heparin effectively reduces postoperative DVT in mobilized colorectal patients, and extended prophylaxis for up to 28 days should be considered in high-risk individuals [33–35]. Inferior vena cava filters are reserved for patients at high thromboembolic risk who cannot receive anticoagulation, as both placement and retrieval carry procedural risks [36, 37]. In patients with colorectal cancer and obesity or severe obesity, anticipation of elevated perioperative risk and risk reduction strategies do not currently include weight loss management during neoadjuvant therapy.

#### Intraoperative challenges: anesthesia

Patients with obesity undergoing major colorectal surgery pose significant anesthetic challenges related to altered physiology, technical complexity, and comorbid disease, resulting in increased perioperative morbidity and mortality [38, 39]. Altered pharmacokinetics and pharmacodynamics require precise dosing to avoid under- or overdosing, as these patients exhibit increased opioid sensitivity and a higher risk of postoperative respiratory depression [40]. Respiratory mechanics are further compromised by decreased functional residual capacity and elevated oxygen consumption, particularly in the Trendelenburg position commonly required for minimally invasive colorectal cancer surgery [41].

Airway management is likewise more challenging in patients with obesity, who often have redundant upper airway soft tissue and obstructive sleep apnea, thereby increasing the risk of difficult ventilation, intubation, and extubation [42]. Higher airway pressures needed for adequate ventilation further increase the risk of pulmonary injury. Regional anesthesia may be technically challenging due to obscured landmarks and increased needle depth in obese body habitus. Positioning and monitoring difficulties also elevate the risk of pressure injury and peripheral nerve damage in obese patients [39]. Collectively, these anesthetic and physiologic challenges underscore the critical need to formally incorporate intentional weight-loss strategies into colorectal cancer prehabilitation pathways to mitigate perioperative risk and optimize surgical outcomes.

#### Intraoperative challenges: oncologic resection and other surgical considerations

Perioperative risk escalates in proportion to the severity of obesity and the burden of associated comorbidities. Visceral adiposity further obscures tissue planes, compromises anatomic delineation, and increases technical complexity, particularly during minimally invasive surgery. Intraoperative challenges may be categorized as general (Table 2), setup-related (Table 3), and procedure-specific technical or perioperative complications (Table 4). Although outcomes are often stratified by BMI < 30 versus ≥ 30, BMIs up to 35 are now common in surgical practice. The greatest technical limitations occur in class II–III obesity, particularly at BMIs > 50, where operative exposure and standard instrumentation may be inadequate. Severe obesity is consistently associated with higher rates of surgical site infection, anastomotic leak, permanent stoma formation, and systemic complications, often necessitating ICU-level care due to prolonged intubation, difficult ventilation, and increased respiratory morbidity (Tables 5, 6) [43–56].

**Table 2:** Risks increased by obesity: general perioperative events

General risks	Examples
General risks:	
Cardiac	Coronary events, congestive heart failure, hypoxia
Pulmonary	Atelectasis, pneumonia, aspiration, edema, effusions, hypoxia
Renal	Renal failure
Diabetes	Decompensation of glucose control, insulin resistance
Thromboembolic	DVT, pulmonary embolism, portal vein thrombosis, arterial ischemia (e.g., stroke)
Pro-inflammatory state	Hypercoagulable state, sepsis-like syndrome
Compartment syndrome	Intraabdominal compartment syndrome, extremity compartment syndrome

**Table 3:** Risks increased by obesity: setup

Setup risk	
Positioning Patient tolerance Pressure injuries	Table and stirrup weight limits, safety and positional stability on the table Tolerance of steep Trendelenburg position, impact on breathability Neuropathy, pressure soars, rhabdomyolysis, extremity ischemia

**Table 4:** Risks increased by obesity: surgery-specific

Surgery-specific risks	Examples
MIS technical challenges:	Primary port placement (Hasson, Veress) Safe port closure (Carter-Thompson) Significant torque on trocars Insufficient tolerance of pneumoperitoneum Inadequate workspace Limited instrument reach Inability to identify anatomical landmarks Challenging dissection (pelvis, vascular stalks) Ability to manage complications (bleeding, air leak) Ability to create a pelvic pouch (IPAA, colonic?) Incomplete specimen Increased risk for long-term need for stoma Prolonged operative times Increased conversion rates
Open technical challenges:	Choice of incision (vertical, horizontal) Combination with panniculectomy Durable fascial closure
Modality-independent challenges:	Collateral injury (ureter, small bowel, spleen etc.) Stoma creation (location, reach, retraction, hernia) Retained foreign objects Surgical site infections Anastomotic leak Prolonged postoperative ileus Hernia formation

**Table 5:** Comparative incidence of SSIs and leaks in obese and non-obese patients (studies with absolute numbers)

Author	Year	No obesity	Obesity BMI ≥ 30	% of patients with obesity	% SSI No obesity	% SSI obesity	% Leak No obesity	% Leak obesity	Comment
Bokey [43]	2014	160	95	37.3	7.5	8.4	2.5	8.3	
Amri [44]	2014	725	301	29.3	4.7	13.3	1.9	1.3	
Chand [45]	2015	204	50	19.7	1.0	2.0	1.0	2.0	
Frasson [46]	2016	1060	42	3.8	12.9	26.2	NR	NR	
Gebauer [47]	2017	7996	1924	19.4	3.7	7.5	7.5	8.3	
Heus [48]	2019	134	272	67.0	7.5	14.3	2.2	4.8	VFA < / ≥ 100
Peacock [49]	2020	372	161	30.2	2.4	6.8	4.3	3.7	
Zhang [50]	2021	308	48	13.5	8.8	12.5	0.3	12.5	
Hannan [51]	2022	73	34	31.8	16.4	29.4	5.5	2.9	
Zhao [52]	2024	1203	210	14.9	1.5	1.4	4.2	5.7	BMI < / ≥ 28
Total		12,235 (12,195)	3137 (3114)		4.5	8.7	5.6	6.7

**Table 6:** Comparative incidence of SSIs and leaks in patients with and without obesity (studies not reporting absolute numbers)

Author	Year	No obesity	Obesity BMI ≥ 30	% of patients with obesity	Subgroups	% SSI No obesity	% SSI Obesity	% Leak No obesity	% Leak obesity	Comment
Smith [53]	2014	8945	3050	25.4		0.9—1.42 × HR	12.0—20.1 HR	NR	NR	Only HR reported
Winfeld [54]	2016	53,743	35,405	39.7	Clean Clean contaminated Contaminated	~ 2—13% ~ 9% ~ 13—14.5%	~ 4—6% ~ 12—13% ~ 16—17%	NR	NR	NEFC*
Docimo [55]	2019	1727	2657	60.6		9—10%	12—20%	NR	NR	BMI < / ≥ 35, NEFC*
Meijs [56]	2019	274,793	113,126	29.2	Open colon: Lap colon:	15.18—17.91% 6.91—8.57%	20.67—32.04% 10.62—17.11%	NR	NR	Colon surgeries only fraction of total N

^*^NEFC numbers extracted from chart

Obesity, especially visceral obesity, adversely affects both short- and long-term colorectal cancer outcomes. It is a recognized risk factor for conversion from minimally invasive to open surgery [57], which increases wound complications and may delay adjuvant therapy. Across surgical platforms, achieving oncologic principles—high vascular ligation and complete mesocolic excision (CME) or total mesorectal excision (TME)—is more technically demanding. In rectal cancer, higher BMI is associated with reduced response to neoadjuvant therapy, inferior TME specimen quality [58], lower sphincter preservation rates [59–61], and increased postoperative morbidity [62–64]. Quantitative imaging metrics, including mesorectal fat area and pelvic dimensions, predict TME complexity and correlate with impaired specimen quality and adverse anastomotic outcomes [65].

Surgical planning in patients with severe obesity must remain grounded in established oncologic principles. Minimally invasive approaches should be employed when technically feasible and oncologically sound. Operative strategy should be individualized according to patient anatomy, tumor characteristics, and physiologic reserve. Robotic platforms have expanded the feasibility of resection in patients with significant visceral adiposity and are associated with lower rates of conversion. However, conversion to an open approach should be considered a prudent intraoperative judgment when required to ensure safety or oncologic adequacy, rather than a procedural failure. Key operative tasks, performance benchmarks, and preferred technical strategies are summarized in Table 7.

**Table 7:** Preferred surgical strategies for specific tasks or benchmarks

Task/benchmark	Preferred strategy for patients with severe obesity
Pelvic dissection	As MIS* as possible, as open as necessary, possible transanal TME
Oncological resection	As MIS* as possible, as open as necessary
Multi-visceral resection	Individualized: MIS* vs open
Simultaneous panniculectomy or VRAM flap	Open, preferably via transverse incision
Creation of pneumoperitoneum	Veress needle → higher incidence of preperitoneal insufflation Hasson → Challenging to find fascia in the depth Primary placement of extraction site with Mini-GelPOINT™
Conversion	Robotic
Anastomotic leak	As MIS* as possible, as open as necessary
Stoma	Pre-operative marking, upper abdomen, possible inside-out construction
Duration of surgery	Open
Estimated blood loss	MIS*
Length of stay (LOS)	MIS*
Wound infection	MIS*
Hernia	MIS*
Adhesions	MIS*

^*^MIS minimally invasive surgery

### Weight loss modalities overview

Despite the substantial evidence demonstrating the adverse impact of obesity—particularly severe obesity—on colorectal cancer (CRC) staging, operative management, and outcomes, contemporary prehabilitation pathways do not incorporate structured weight-loss interventions for patients with obesity. This omission largely reflects concern regarding potential delays in oncologic therapy, and to date, no major surgical society guidelines formally endorse integration of weight-loss strategies during neoadjuvant treatment in this population.

Given the magnitude of perioperative and oncologic risk, reconsideration of this paradigm is warranted. The subsequent sections of this manuscript outline evidence-informed weight-loss strategies that may be integrated into a coordinated model of prehabilitation, neoadjuvant therapy, and surgical planning for patients with concurrent severe obesity and CRC. These approaches encompass nonoperative interventions—including dietary modification, exercise optimization, and pharmacotherapy—as well as metabolic and bariatric surgical options within a synchronized multidisciplinary framework.

#### Non-surgical weight loss options and impact on colorectal cancer outcomes

##### Lifestyle modifications

Dietary and exercise interventions offer general health benefits. However, sustained weight loss is typically limited to 2–10% of total body weight and is frequently followed by weight regain. Consequently, lifestyle modification alone is unlikely to achieve clinically meaningful weight optimization [66]. Calorie-restricted liquid diets have been associated with reduced colorectal tissue expression of Ki-67, a proliferation marker, and decreased insulin levels implicated in CRC-related inflammatory pathways [67] further supporting moving forward with synchronized weight loss and CRC management with the goal of improved oncologic outcomes.

Pre-operative very low-energy diets (VLEDs) have also been shown to significantly reduce mesorectal fat before pelvic colorectal surgery [68]. A retrospective cohort study reported that VLED use was associated with lower 30-day postoperative morbidity—including respiratory, cardiovascular, and wound complications—and fewer ICU admissions, without a significant reduction in gastrointestinal complications [69]. The CARE Feasibility Randomized Clinical Trial compared VLED with usual care before CRC resection and demonstrated greater preoperative weight loss (6.1 kg vs 1.8 kg; 95% CI, 2.7–5.8 kg), but no difference in 30-day postoperative complication rates [70, 71].

##### Medical weight loss options

Anti-obesity medications (AOMs) are increasingly used as standalone therapy or adjuncts to metabolic and bariatric surgery [72]. Currently approved agents include orlistat, phentermine/topiramate, naltrexone/bupropion, and glucagon-like peptide-1 (GLP-1) receptor agonists. Their mechanisms include reduced fat absorption (orlistat), appetite suppression and craving reduction (phentermine/topiramate, naltrexone/bupropion), and delayed gastric emptying with appetite modulation (GLP-1 agonists). Population-based SEER–Medicare data demonstrate an inverse association between AOM use and colorectal cancer (CRC) risk (OR 0.86; 95% CI 0.80–0.92) [73].

Adverse effects are common and are a leading cause of discontinuation of medical weight therapy (Table 8). These are predominantly gastrointestinal, including nausea, vomiting, constipation, diarrhea, and gastroparesis. Delayed gastric emptying and aspiration risk have prompted anesthesia societies to recommend holding GLP-1 agonists before general anesthesia. However, recommended discontinuation intervals vary widely from 1 day to 1 week before elective surgery [74]. Rare but serious complications, including pancreatitis, bowel obstruction, and bowel perforation, have been reported [75–79].

**Table 8:** Reported side effects of GLP-1 agonists for obesity

Study	Year	Drug(s) studied	GI side effects (Common)	Serious GI events	Notes
Filippatos [78]	2015	Exenatide, Liraglutide, others	Nausea (up to 50%), Diarrhea, Constipation, Abdominal pain, Dyspepsia	Pancreatitis concerns from animal models and case reports	Meta-analyses inconclusive; case reports exist
Wharton [76]	2021	Semaglutide 2.4 mg (weekly)	Nausea (43.9%), Diarrhea (29.7%), Vomiting (24.5%), Constipation (24.2%)	Few discontinuations (4.3%); mostly mild/moderate	Events peaked during dose escalation and were usually transient
Sodhi [79]	2023	Semaglutide, Liraglutide	Pancreatitis (HR 9.09), Bowel obstruction (HR 4.22), Gastroparesis (HR 3.67)	Rare but significantly elevated risks of pancreatitis, obstruction, gastroparesis	Compared with bupropion-naltrexone, higher hazard ratios noted
Gudzune [77]	2024	Liraglutide, Semaglutide, Tirzepatide	Nausea (28–44%), Diarrhea (21–30%), Constipation (11–24%)	Generally low incidence of serious events	Review of various drug classes; focuses on tolerability range
Moiz [75]	2025	Liraglutide, Semaglutide, Tirzepatide	Nausea, Vomiting, Diarrhea, Constipation (up to 80% incidence)	Serious GI AEs ‚ < 3.5%; Pancreatitis < 2%	Wide variability in AE reporting; based on 26 RCTs

When tolerated, GLP-1 agonists are associated with improved cardiovascular outcomes and reduced all-cause morbidity and mortality [80, 81]. Pharmacologic weight loss has been successfully used to optimize patients before complex abdominal wall reconstruction [82]. Treatment with GLP-1 agonists for 8–12 months in patients with severe obesity produced safe and effective weight reduction and enabled surgical eligibility, although no reduction in postoperative complications or length of stay was observed [83].

To date, no studies have specifically evaluated GLP-1 agonists for preoperative weight loss before colorectal cancer surgery. Long-term trials of semaglutide (208 weeks) demonstrate a mean total weight loss of 10.2% with a 7.7-cm reduction in waist circumference, with most weight loss occurring within the first 26 weeks [94]. Tirzepatide, a dual GLP-1 and glucose-dependent insulinotropic polypeptide receptor agonist, achieved 21% total body weight loss at 72 weeks [66]. Both agents require monitored dose escalation with concurrent dietary counseling to preserve protein intake. Semaglutide is initiated at 0.25 mg weekly for 4 weeks and increased every 4 weeks to 0.5 mg, 1.0 mg, and 1.7 mg, with a target maintenance dose of 2.4 mg weekly [95]. Tirzepatide is initiated at 2.5 mg weekly for 4 weeks and escalated every 4 weeks to 5 mg, 10 mg, and 15 mg, targeting a 15-mg maintenance dose by week 20 [96].

The beneficial effect of nonsurgical weight loss on CRC risk requires further validation in large, long-term cohort studies [67]. Limited insurance coverage for pharmacologic therapy may be a hurdle and should be considered when evaluating patients for synchronized weight loss and CRC treatment. Durable and effective weight reduction strategies remain essential for mitigating CRC risk and improving both oncologic and surgical outcomes.

#### Surgical weight loss options and impact on colorectal cancer outcomes

Despite the expanding use of pharmacologic weight loss therapies, metabolic and bariatric surgery (MBS) remains the most effective and durable treatment for obesity. Sleeve gastrectomy and Roux-en-Y gastric bypass achieve approximately 25% and 30% total weight loss at 12 months, respectively [66]. Obesity is associated with a 30–70% increased risk of colorectal cancer (CRC) [97], with a stronger effect observed in early-onset disease (OR 1.82; 95% CI 1.62–2.04; *p* < 0.001). Bariatric surgery is associated with a 36–54% reduction in CRC incidence [98–101]. Similar risk reductions are observed for colon (RR 0.75; 95% CI 0.53–1.06) and rectal cancer (RR 0.74; 95% CI 0.48–1.15). Compared with the general population, CRC risk is comparable after MBS, whereas patients with obesity who do not undergo MBS exhibit a 34% higher risk [93].

Prior bariatric surgery is associated with fewer intraoperative and postoperative complications after colorectal surgery, as well as reduced healthcare utilization and shorter hospital length of stay (LOS) [93, 102, 103]. Among MBS procedures, sleeve gastrectomy appears to confer greater risk reduction than Roux-en-Y gastric bypass (RR 0.48 vs. 0.64) [104]. These benefits may be driven by rapid changes in body composition, particularly reductions in visceral adiposity, which decreased by 15–33% within three months after sleeve gastrectomy [105–107].

Given the technical challenges and increased morbidity associated with severe obesity, preoperative optimization for major abdominal surgery—including hernia repair, hysterectomy, transplantation, and elective colorectal surgery—should include consideration of MBS [108–110]. Limited evidence suggests that MBS performed as a bridge to ventral hernia repair is safe when the hernia is left untreated, with low complication rates [111]. In patients with end-stage organ failure, particularly liver disease requiring transplantation, multidisciplinary expert consensus supports the safe integration of MBS in transplant candidates. Weight loss prehabilitation appears to improve transplant eligibility, reduce contraindications, and lower perioperative complication rates to approximately 2–5% [112–114] Despite growing evidence supporting metabolic and bariatric surgery as a safe and effective preoperative optimization strategy across multiple high-risk surgical populations, a comparable integration of structured weight-loss management remains notably absent in colorectal cancer care, perpetuating a significant disparity in treatment paradigms for patients with severe obesity.

#### Pre-operative weight loss in patients with colorectal cancer

Emerging evidence suggests that prior bariatric surgery is associated with reduced intraoperative and postoperative complications following colorectal resection, as well as decreased healthcare utilization and length of stay. These findings support consideration of structured weight-loss prehabilitation within the colorectal cancer (CRC) care pathway [115]. In a series of 37 patients with malignancy deemed inoperable or high-risk due to severe obesity, metabolic and bariatric surgery (MBS) facilitated oncologic optimization in 83.8% of patients—including all individuals with CRC—enabling progression to definitive resection [115]. These data highlight the potential role of MBS and other weight-loss interventions within a coordinated, multidisciplinary oncologic strategy.

The current literature remains limited, with one case series describing patients with early-stage CRC who underwent MBS prior to colorectal resection, achieving substantial weight loss without evidence of disease progression [116]. Accordingly, weight-loss prehabilitation may be appropriate when it does not compromise oncologic timelines. As obesity and its related comorbidities represent modifiable risk factors, preoperative optimization of glycemic control, nutritional status, and physical conditioning may contribute to improved postoperative outcomes.

MBS may be particularly suitable for patients with indolent tumor biology or in circumstances where severe obesity renders resection technically prohibitive. Even in higher-risk tumors, metabolic optimization may favorably influence systemic inflammation and the tumor microenvironment. However, implementation remains challenging due to insurance authorization requirements and the need for comprehensive multidisciplinary evaluation, which may conflict with the time-sensitive, multimodal nature of CRC treatment. For colon cancer, concerns center on delaying definitive surgery; for rectal cancer, on postponing initiation of neoadjuvant therapy [93]. Early identification of obesity is therefore essential, particularly given its stronger association with early-onset CRC [92]. Selected high-risk patients who might otherwise be denied surgical intervention due to severe central adiposity could be considered for bariatric evaluation as practice standards evolve.

Balancing perioperative risk reduction against potential delays in oncologic care remains central. Modeling studies suggest that, for stage I–III CRC, delaying surgery beyond 120 days may increase 5-year mortality from 25 to 37% [117, 118]. Notably, these analyses do not account for concurrent neoadjuvant therapy. In rectal cancer—where treatment frequently includes 6–9 months of chemoradiation and consolidation chemotherapy—this interval may provide a window for targeted metabolic optimization prior to resection. The optimal sequencing of weight-loss intervention relative to neoadjuvant therapy remains undefined and warrants prospective investigation [118].

Although anti-obesity pharmacotherapy offers a less invasive alternative, comparative data demonstrate inferior efficacy relative to MBS. Bariatric surgery achieves significantly greater total weight loss than GLP-1-based therapy (28.3% vs 10.3%; *p* < 0.001) and confers greater reductions in obesity-related comorbidities with favorable long-term cost implications [119]. Nevertheless, until barriers to MBS access are addressed, many patients with CRC will remain limited to medical weight-loss strategies during the preoperative period.

### Limitations of current guidelines

The 2022 American Society for Metabolic and Bariatric Surgery (ASMBS) guidelines acknowledge metabolic and bariatric surgery (MBS) as an appropriate bridge to selected interventions, including joint arthroplasty, abdominal wall hernia repair, and organ transplantation (Table 9) [120]. However, two important limitations warrant consideration in the context of colorectal cancer (CRC) management. First, the guidelines explicitly state that “there are no data to support the practice of insurance-mandated preoperative weight loss,” characterizing such requirements as discriminatory, arbitrary, and scientifically unsupported, and noting that they may contribute to treatment delays and progression of obesity-related comorbid disease. Second, although no absolute contraindications to MBS are identified, active cancer treatment is generally regarded as a relative contraindication [121]. This position may inadvertently exclude patients with concurrent severe obesity and malignancy from consideration of surgical metabolic optimization, even when obesity itself meaningfully increases operative and oncologic risk.

**Table 9:** Summary of the 2022 guidelines from the American Society of Metabolic and Bariatric Surgery [120]

Accepted indications:	• Recommended for patients with body mass index ≥ 35 kg/m^2^, regardless of presence, absence, or severity of comorbidities • Consider for patients with metabolic syndrome and body mass index between 30 and 34.9 kg/m^2^ • Recommended for patients of Asian descent with body mass index ≥ 27.5 kg/m^2^ • No formal age limit in older populations but careful patient selection (with an emphasis on frailty assessment) is suggested • Bridge to other therapy - Joint arthroplasty - Abdominal wall hernia repair - Organ transplantation
Relative indications	• Compensated cirrhosis (risk remains small (< 1%) • Congestive heart failure
Relative contraindications	• Severe heart failure • Unstable coronary artery disease • End-stage lung disease • **Active cancer treatment** • Drug/alcohol dependency • Impaired intellectual capacity

Timeliness of CRC treatment is widely emphasized; however, the evidence defining an acceptable interval to definitive therapy remains limited and heterogeneous. For colon cancer, no consensus guidelines establish a clear maximal timeframe to surgery [122]. Proposed benchmarks—such as operative intervention within 28 days of consultation—are supported by limited data [123, 124]. Several population-based analyses demonstrate no adverse oncologic impact with modestly longer intervals [125–127], whereas one meta-analysis suggests worsened outcomes beyond 45 days [128]. For rectal cancer, the National Accreditation Program for Rectal Cancer recommends initiation of definitive treatment—including surgery, systemic therapy, radiotherapy, or multimodal management—within 60 days of diagnosis [129]. Nevertheless, the evidence underpinning this threshold remains inconsistent and of variable quality [130, 131].

### Moving forward—synchronizing colorectal cancer and weight loss management

The existing literature describing metabolic and bariatric surgery (MBS) as a bridge to colorectal surgery remains sparse, limited largely to isolated case reports documenting integration within the oncologic care pathway. To address this gap, the following section presents illustrative cases from our working group that demonstrate a potential “window of opportunity” for synchronizing weight-loss prehabilitation with colorectal cancer (CRC) management.

In most instances, patients initially pursued medical weight-loss therapy, as insurance authorization requirements and the comprehensive multidisciplinary evaluation necessary for MBS often impose extended timelines. Consequently, nonsurgical interventions frequently represent the only immediately accessible option, highlighting the present reliance on pharmacologic and lifestyle-based strategies during the preoperative period. These practical constraints underscore the need for structured pathways that align metabolic optimization with time-sensitive oncologic treatment.

**Fig. 1:**
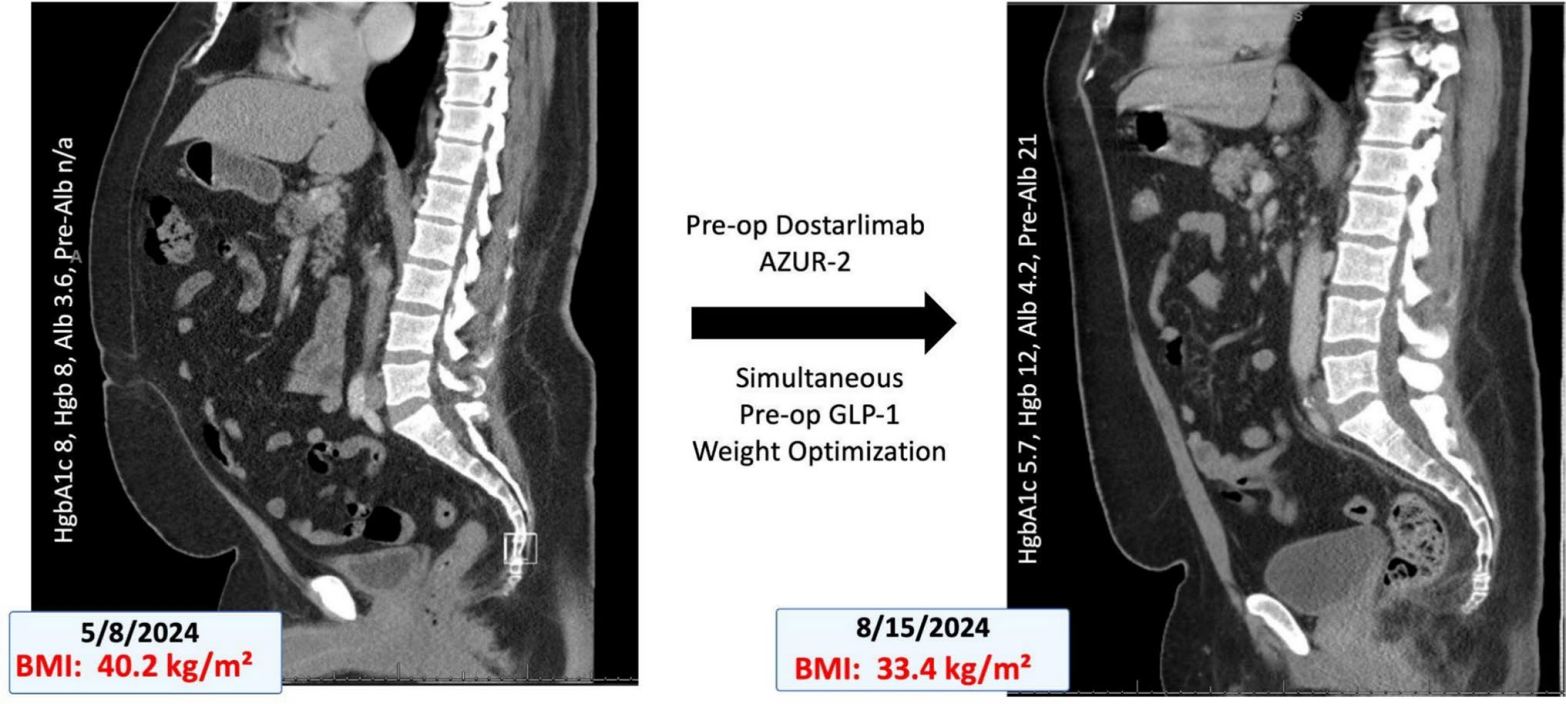
Cross-sectional imaging of visceral obesity before and after synchronous management of a dMMR colon cancer patient with morbid obesity

Case Example 1: Medically supervised synchronous weight loss using GLP-1 inhibition was achieved in a patient with mismatch repair-deficient (dMMR) colon cancer enrolled in the AZUR-2 trial and randomized to three months of neoadjuvant immunotherapy (dostarlimab) [132]. The patient lost 22.3 kg, with BMI decreasing from 40.2 at diagnosis to 33.5 at surgery, accompanied by a marked reduction in visceral adiposity (Fig. 1). Nutritional status was preserved, with serum albumin 4.2 g/dL and prealbumin 21 mg/dL on the day of surgery. Final pathology revealed ypT2N1a disease with one positive lymph node. The patient completed adjuvant immunotherapy per protocol without delay and remains without evidence of disease more than one year after treatment completion.

Case Example 2: A GLP-1 receptor agonist was administered during total neoadjuvant therapy (TNT) in a patient with T3aN + mismatch repair-proficient (pMMR) rectal cancer and a baseline BMI of 59. Medically supervised synchronous weight loss reduced BMI from 59 to 53 by the time of surgery. The patient underwent abdominoperineal resection with permanent colostomy with negative margins and ypT2N0 disease. The patient remains disease-free more than 1 year postoperatively.

Case Example 3: A 41-year-old man with a BMI of 67.48 kg/m^2^ was diagnosed with cT3cN2 low rectal adenocarcinoma. After completing neoadjuvant chemoradiation, he was referred for surgical evaluation. Severe obesity raised concern for the feasibility and safety of oncologic resection due to anticipated anesthetic and ventilatory challenges, difficulty with pelvic dissection and reconstruction, ostomy reach, and increased risks of wound complications, surgical site infection, and hernia formation.

The patient was referred for metabolic and bariatric surgery (MBS) as part of preoperative optimization. Sleeve gastrectomy was selected to facilitate improved ostomy creation and management and to reduce postoperative colorectal surgical risk. As he was not receiving active chemotherapy and had no contraindications, bariatric surgery was expedited during this “window of opportunity.”

**Fig. 2:**
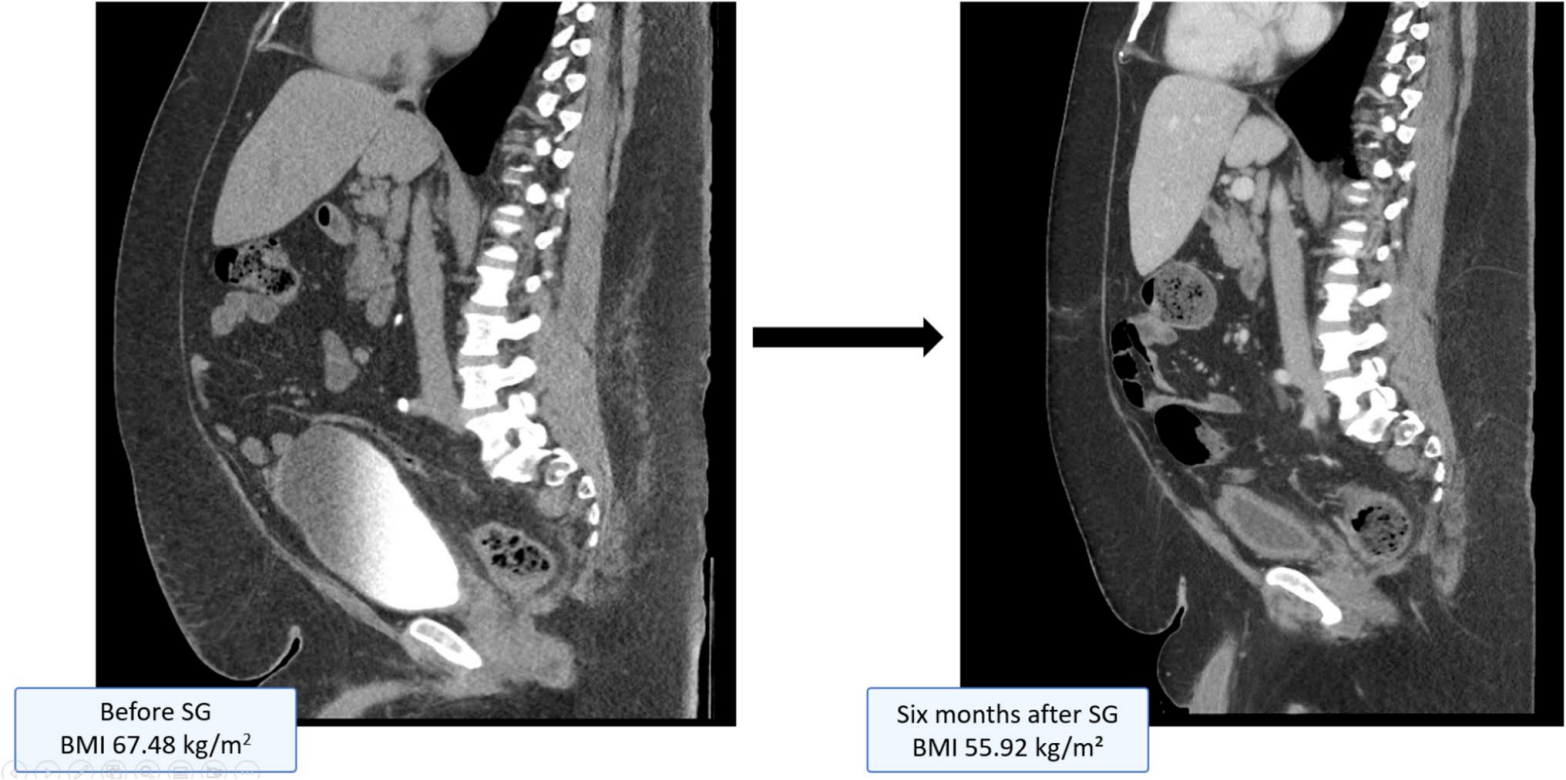
Cross-sectional imaging before and after sleeve gastrectomy of a patient with a cT3cN2 low rectal adenocarcinoma and morbid obesity

The post-sleeve gastrectomy course was uncomplicated. The patient achieved a nadir BMI of 51.39 kg/m^2^ at 15 months. Eight months after bariatric surgery, he underwent a minimally invasive low anterior resection with diverting loop ileostomy, followed by an ostomy reversal 3 months later. He remains well and disease-free at 4 years (Fig. 2). Before and after imaging demonstrates a marked reduction in visceral adiposity after weight loss prehabilitation.

#### Windows of opportunity warranting further investigation

**Fig. 3:**
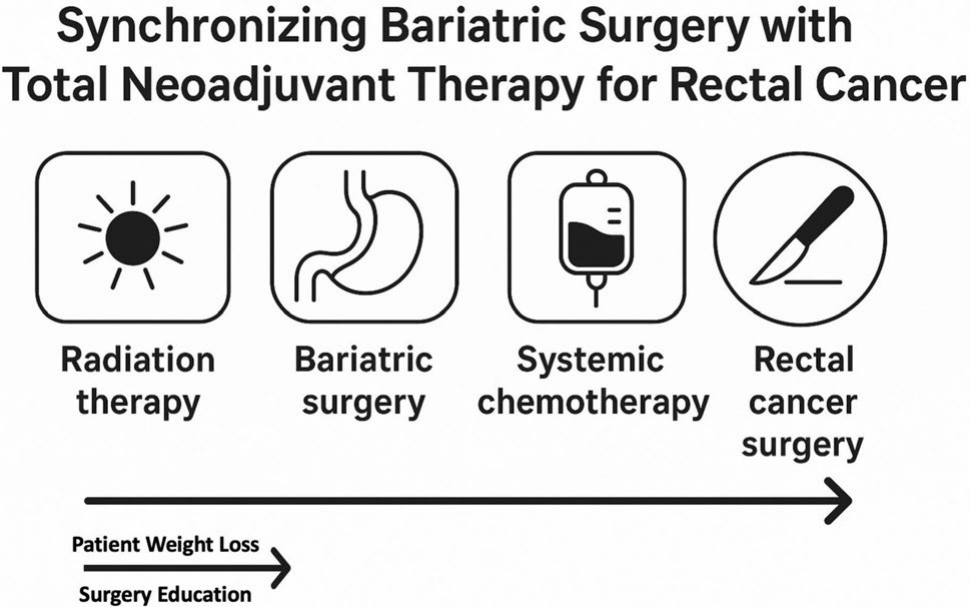
Opportunity for synchronizing bariatric surgery with TNT for pMMR rectal cancer

**Fig. 4:**
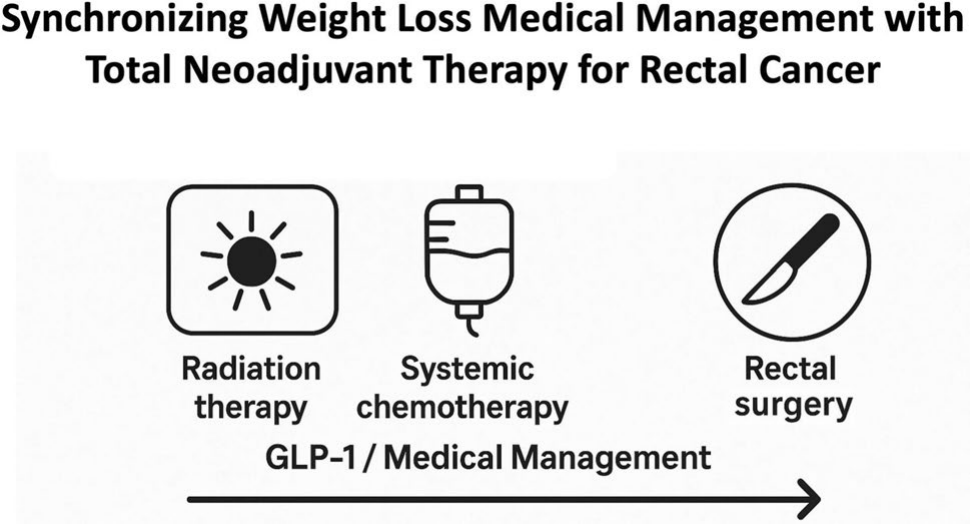
Opportunity for synchronizing medical weight loss with TNT for pMMR rectal cancer

Management of locally advanced mismatch repair–proficient (pMMR) rectal cancer with total neoadjuvant therapy (TNT) creates a prolonged interval before planned surgical resection. When TNT begins with radiation and radio-sensitizing chemotherapy, a natural gap exists between completion of radiation and initiation of systemic chemotherapy during which bariatric surgery could be considered (Fig. 3). During long-course radiation, patient education, psychiatric evaluation, nutritional counseling, and weight-loss instruction may be conducted concurrently, enabling synchronization of obesity management with oncologic therapy. Medically supervised weight-loss programs for severe obesity may likewise be implemented throughout the TNT period before surgery (Fig. 4).

**Fig. 5:**
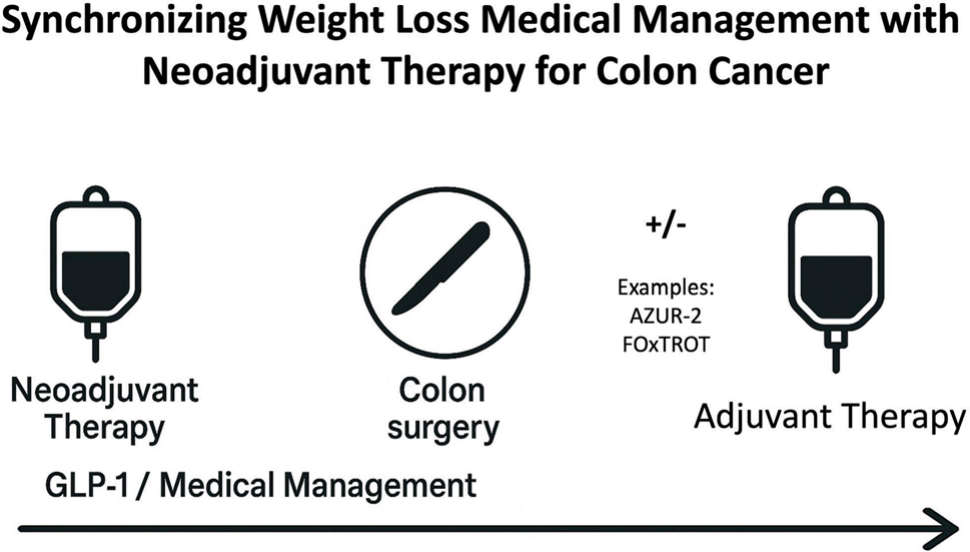
Opportunity for synchronizing medical weight loss with neoadjuvant therapy for colon cancer

Similar opportunities may exist for patients with severe obesity and colon cancer. Neoadjuvant systemic therapy is being evaluated in trials such as FOxTROT and AZUR-2, which assess the initiation of chemotherapy before surgical resection (Fig. 5). Pre-operative optimization—encompassing nutritional support, anemia correction, and physical conditioning—is already standard practice in colorectal surgery. Integrating structured weight-loss prehabilitation into this framework represents a logical extension and warrants evaluation in prospective, controlled clinical trials.

#### Multidisciplinary team engagement

Patients with colorectal cancer (CRC) and severe obesity represent a particularly complex clinical population [133, 134]. Achieving coordinated weight loss and optimal oncologic outcomes requires strong multidisciplinary collaboration. Integration of colorectal surgery, medical oncology, and radiation oncology improves care coordination and treatment completeness [135]. Institutions using formal multidisciplinary CRC models demonstrate more accurate staging, greater access to multimodal therapy, and approximately 20% reductions in all-cause and cancer-specific mortality compared with single-specialty care [135, 136]. Early multidisciplinary alignment facilitates shared assessment of tumor biology, disease stage, and patient-specific factors.

In patients with severe obesity, multidisciplinary evaluation must also address obesity-related considerations, including chemotherapy dosing during weight loss, radiation planning and positioning, management of hypertension and diabetes, anesthetic risk, technical operative feasibility, and suitability for minimally invasive surgery. Collaborative care has been associated with improved survival: strong surgeon–oncologist partnerships reduce all-cause and cancer-specific mortality [136], and multidisciplinary cancer conferences prolong overall survival by broadening clinical perspectives and optimizing care delivery [137].

Accordingly, multidisciplinary coordination among colorectal surgery, medical oncology, and radiation oncology is essential in the management of patients with concurrent CRC and severe obesity. Such a team-based framework facilitates individualized, guideline-concordant multimodal therapy and is associated with improved short-term perioperative and oncologic outcomes, as well as enhanced long-term survival. Within this context, deliberate identification of potential “windows of opportunity” for weight-loss prehabilitation should be incorporated into routine multidisciplinary discussion. The integrated management pathways outlined in Figs. 3, 4 and 5 provide a proposed framework for synchronized care and should serve as a structured component of case review and treatment planning.

#### Patient engagement

Patient education is essential for successful weight loss interventions and may be delivered individually or in group settings with comparable efficacy [138]. Randomized studies of both anti-obesity medications and bariatric surgery demonstrate improved weight loss when dietary education and ongoing multidisciplinary support are incorporated. Emphasizing improvements in obesity-related comorbidities, such as obstructive sleep apnea and hypertension, in addition to weight reduction may enhance patient motivation. Weight management counseling should avoid stigmatizing language and implicit bias. Many patients with obesity report negative healthcare experiences that discourage future care-seeking. Use of person-first language (e.g., “patient with obesity”) and provision of appropriate equipment (gowns, chairs, examination tables) promote a more inclusive clinical environment [139].

Weight loss targets should be realistic and evidence-based [140]. Discussing expected total weight loss helps patients contextualize outcomes from each intervention. Patients should be counseled that weight loss occurs gradually and variably over time to prevent discouragement during plateaus. Even modest early weight loss can yield meaningful physiological benefits, as observed in early improvements in type 2 diabetes after metabolic and bariatric surgery. Collaboration with a multidisciplinary weight management team optimizes education, goal setting, and long-term adherence.

#### Ethical considerations

Management of severe obesity in patients with colorectal cancer raises several ethical challenges, including:

- Whether oncologic treatment may be withheld based on obesity-related risk
- Whether preoperative weight loss can be mandated and if such requirements prioritize institutional policy over patient welfare
- The extent to which insurers should be obligated to cover preparatory pharmacologic or surgical interventions
- Informed consent obligations regarding elevated perioperative and oncologic risk for patients and families
- Whether cases involving conflicting guidelines, feasibility concerns, and patient safety should require institutional or state-level ethical review

#### Financial considerations

Care of high-risk patients with severe obesity entails longer operative times and greater technical complexity, raising concerns about inadequate reimbursement under current fee structures. Standard compensation often fails to reflect case complexity, and modifier 22 adjustments may be insufficient. If unaddressed, these mismatched risks may contribute to financial pressures to influence patient selection rather than clinical need. Coverage for medical weight-loss therapy and bariatric surgery in patients with severe obesity is likewise inconsistent and frequently delayed. Further evaluation of synchronized weight-loss interventions alongside colorectal cancer care may inform future guidelines and help reduce financial and access barriers to this integrated approach.

## Conclusions

Obesity and severe obesity represent an increasingly important and underrecognized modifier of colorectal cancer (CRC) care. Severe obesity adversely influences diagnostic accuracy, perioperative risk, technical feasibility of oncologic resection, and long-term outcomes. Despite these effects, current guidelines provide limited or prohibitive direction for managing obesity within CRC treatment pathways, leaving clinicians to navigate competing oncologic urgency, surgical risk, and systemic barriers without evidence-based support.

Emerging data and clinical experience suggest that pharmacologic and surgical weight-loss strategies can meaningfully improve operative conditions, anesthetic tolerance, and postoperative outcomes. Weight loss may also positively modify the tumor microenvironment and systemic risk profile. When thoughtfully integrated into neoadjuvant and preoperative windows of opportunity, weight-loss prehabilitation may enhance both technical and oncologic success without necessarily compromising cancer timeliness.

Implementation of such strategies must balance the urgency of cancer therapy with patient safety and require careful multidisciplinary coordination, ethical oversight, and equitable access to care. These considerations highlight the need for prospective, controlled trials and formal guideline development to define appropriate patient selection, timing, and modality of intervention. Professional societies and collaborative research networks are encouraged to lead efforts to establish evidence-based standards for synchronized weight optimization and CRC treatment. Doing so offers a unique opportunity to improve surgical quality, reduce complications, and narrow the growing disparity between obesity and cancer outcomes—transforming obesity from a fixed liability into a modifiable therapeutic target within modern colorectal cancer care.

## Abbreviations

AOM: Anti-obesity medications
BMI: Body mass index (weight in kg divided by squared height in meters)
CRC: Colorectal cancer
GLP-1: Glucagon-like peptide-1
MBS: Metabolic and bariatric surgery
RYGB: Roux-en-Y gastric bypass
SG: Sleeve gastrectomy
